# Supplementary material for: Complexome profiling of the Chlamydomonas psb28 mutant reveals TEF5 as an early PSII assembly factor
Source: Plant Cell. 2025 Mar 17;37(6):koaf055. doi: 10.1093/plcell/koaf055 (PMC12164590; doi:10.1093/plcell/koaf055)
Supplement: koaf055_Supplementary_Data [file koaf055_supplementary_data.zip › psb28_interactive.zip!/plots/intensity/qualityPlots/peptides/PDX1_Cre15.g643550.t1.2.html]

### Cre15.g643550.t1.2

Trivial name: PDX1  
SeqCoverage [median]: 28.00  
Mass: 31.54 kDa  
  
 

| Sequence | MissedCleavage | Mass [Da] | wtIntensity | muIntensity | Score |
| --- | --- | --- | --- | --- | --- |
| AAVTIPVMAK | 0 | 999.58 | 541.51 | 1096.60 | 75.11 |
| EIRAPVELVR | 1 | 1180.69 | 0.00 | 880.57 | 42.34 |
| IAEEAGAVAVMALER | 0 | 1528.79 | 0.00 | 1288.47 | 66.26 |
| IAEGAAMIR | 0 | 930.50 | 1686.24 | 666.15 | 112.17 |
| MSDPTMIK | 0 | 921.43 | 349.98 | 404.12 | 86.79 |
| SSGTFAVK | 0 | 795.41 | 535.93 | 0.00 | 89.14 |
| VGLAQMLK | 0 | 858.50 | 0.00 | 473.00 | 86.09 |
|  |  |  | 3113.7 | 4808.9 |  |
